# Soft Gripping Fingers Made of Multi-Stacked Dielectric Elastomer Actuators with Backbone Strategy

**DOI:** 10.3390/biomimetics9080505

**Published:** 2024-08-21

**Authors:** Armin Jamali, Robert Knoerlein, Dushyant Bhagwan Mishra, Seyed Alireza Sheikholeslami, Peter Woias, Frank Goldschmidtboeing

**Affiliations:** 1 Cluster of Excellence livMatS @ FIT—Freiburg Center for Interactive Materials and Bioinspired Technologies, University of Freiburg, 79110 Freiburg, Germanydushyantbmishra@gmail.com (D.B.M.); sheikha@tf.uni-freiburg.de (S.A.S.); woias@imtek.uni-freiburg.de (P.W.); fgoldsch@imtek.uni-freiburg.de (F.G.); 2 Laboratory for the Design of Microsystems, Department of Microsystems Engineering (IMTEK), University of Freiburg, 79110 Freiburg, Germany

**Keywords:** soft grippers, dielectric elastomer actuators, soft robotics, bending actuators, backbone strategy

## Abstract

Soft grippers, a rapidly growing subfield of soft robotics, utilize compliant and flexible materials capable of conforming to various shapes. This feature enables them to exert gentle yet, if required, strong gripping forces. In this study, we elaborate on the material selection and fabrication process of gripping fingers based on the dielectric elastomer actuation technique. We study the effects of mixing the silicone elastomer with a silicone thinner on the performance of the actuators. Inspired by nature, where the motion of end-effectors such as soft limbs or fingers is, in many cases, directed by a stiff skeleton, we utilize backbones for translating the planar actuation into a bending motion. Thus, the finger does not need any rigid frame or pre-stretch, as in many other DEA approaches. The idea and function of the backbone strategy are demonstrated by finite element method simulations with COMSOL Multiphysics^®^ 6.5. The paper describes the full methodology from material choice and characterization, design, and simulation to characterization to enable future developments based on our approach. Finally, we present the performance of these actuators in a gripper demonstrator setup. The developed actuators bend up to 68.3° against gravity, and the gripper fingers hold up to 10.3 g against gravity under an actuation voltage of 8 kV.

## 1. Introduction

Soft grippers are end-effectors that use flexible and compliant materials to grasp and manipulate objects. Compared to traditional rigid grippers, they perform advanced grasping tasks and manipulate a larger variety of objects [1,2,3,4,5]. They have promising applications in multiple fields, including crop harvesting, biological sampling on deep reefs, medical instrumentation, surgery, and the development of artificial hands [6,7,8,9]. A review paper by Shintake et al. (2018) provides a comprehensive background and examples of soft robotic grippers since 1978 [1].

**Figure 1:**
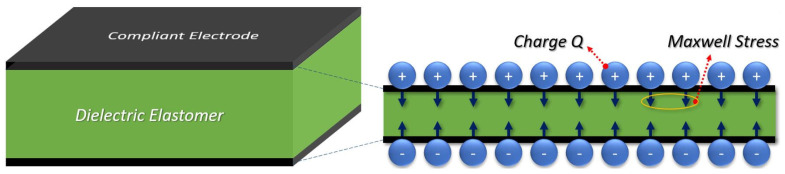
Actuation mechanism of a DEA. The Maxwell stress caused by the high voltage compresses the dielectric in thickness and expands its area.

Soft grippers employ various actuation methods and techniques such as pneumatic actuation [10], shape-memory polymers [1], low-voltage ionic electroactive soft actuators [11,12], or electro adhesion-based DEAs [13]. In this paper, we present a soft gripper that consists of bending dielectric elastomer actuators (DEAs). DEAs are recognized as a type of artificial muscle within the realm of electroactive polymer actuators driven by electrostatic forces. DEAs transform electrical stimulation into mechanical actuation. In their fundamental configuration, DEAs consist of a thin layer of non-conductive dielectric elastomer sandwiched between two compliant electrode layers [14]. Figure 1 illustrates the actuation mechanism of such a DEA with one active layer. When a high voltage is applied, the resulting electrostatic attraction between the electrode layers generates a compression stress, known as Maxwell stress. This causes the elastomer to contract in thickness while expanding in area. In this deformation, it can be approximated that the actuator maintains a constant volume, given a Poisson number close to 0.5 for many of the elastomers in use [15]. Gupta et al. (2019) reviewed soft robots based on DEAs [16].

Though DEAs are often referred to as artificial muscles, Figure 1 already visualizes two important differences from biological muscles. While muscle fibers act only in the direction of the fiber, DEAs produce a two-dimensional in-plane stress pattern. Furthermore, DEAs expand in area while biological muscles contract. A method inspired by nature is used to achieve muscle-like behavior: a skeleton made of stiffer materials is used to direct the force and suppress unwanted motion. It has been presented that a structure similar to a skeleton can be used to modify the actuation behavior of DEAs [17]. In our study, we use a backbone structure as an antagonist to the DEA to convert the stretching of the DEA into bending and stiffening bars inside the backbone structure to suppress unwanted bending in the perpendicular direction.

Typically, very high bond (VHB) acrylic films from 3M are employed to construct DEAs because of their large voltage-induced strains [18,19]. By pre-stretching, the electromechanical instability is suppressed and higher actuation voltages can be applied to the actuator until electrical breakdown occurs. However, the pre-stretched elastomer needs a rigid frame to keep it in place, which limits the flexibility and adaptability of the DEAs. Furthermore, because the elastomer needs to be pre-stretched, fabricating and mass-producing DEAs becomes more complex and difficult [16,20,21]. Moreover, VHB tapes only come in a limited choice of thickness. Due to VHB’s high viscoelasticity, the fabricated DEAs drop their strain over frequency very fast [22]. Silicones, on the other hand, outperform acrylics in response speed due to the significantly lower viscoelasticity [23]. More importantly, uncured silicone elastomers are in a liquid form, which can be deposited in almost every required thickness and shape. However, the strains of silicones are usually much lower than those of acrylic elastomers [24]. Michel et al. have presented a thorough comparison between silicone and acrylic elastomers as dielectric materials [25].

The other important element for constructing DEAs is compliant electrodes, which must be highly conductive, compliant, and capable of surviving a large number of actuations. The electrodes should exhibit a slow rate of degradation, along with high stability and reliability. One of the most commonly used materials for creating compliant electrodes is carbon grease, which is a mixture of carbon black particles suspended in silicone oil. It can be easily applied to the surface of an elastomer and provides strong adhesion while minimally affecting the actuation strain [16]. Although carbon grease remains conductive at high actuation strains, it is difficult to pattern and integrate carbon grease-based soft actuators with other devices since carbon grease is a viscous fluid [26]. A general technique to improve the actuation performance of DEAs is stacking multiple layers of actuation units, which is referred to as “multi-stacking”. However, it is challenging to implement this technique with carbon grease electrodes. Furthermore, the oil-based emulsifier within the carbon grease may diffuse into the dielectric membrane, potentially causing a short circuit. Consequently, the lifetime of DEAs with carbon grease electrodes is relatively short [27].

Beyond optimizing the material properties, the design of effective actuation mechanisms is paramount for effective DEAs. In their basic form, as shown in Figure 1, DEAs execute planar or 2D actuation, necessitating mechanisms for translating these motions into more intricate 3D actions such as bending or twisting. Wang et al. (2021) provide an example of a DEA soft gripper with such a translation mechanism. They optimized an actuation mechanism to convert planar actuation into gripping force using Bezier curves utilizing conventional VHB 4910 acrylic elastomer tape and carbon grease electrodes. Ultimately, their actuators achieved a gripping force of 100.2 mN [28]. Another prominent study by Shian et al. (2015) implemented Vinyl fibers in planar actuators made of acrylic elastomers (VHB 9473PC, 3M) to direct the bending direction of their dielectric elastomer grippers [29]. Duduta et al. (2016) also focus on fabricating multi-layered dielectric elastomers. They combined UV-curable acrylic polymers and single-wall carbon nanotube network electrodes to fabricate ultra-thin actuators to achieve different actuation modes [30]. Last but not least, Yang et al. (2023) presented a muscle-inspired soft robot with bending, locomotion, load carrying, and gripping abilities using VHB 4910 dielectric elastomers and carbon grease. They pre-stretched the actuators (400% × 400%) over a thick PET film [31].

In this study, we demonstrate a versatile fabrication method for DEA gripping fingers that allows the implementation of a backbone structure to mimic the behavior of real fingers that can grab and lift objects without the need for a pre-stretch and rigid frame. To achieve this, we first characterize the corresponding electromechanical properties of the elastomer and electrode materials used for fabricating gripping fingers. Then, we elaborate on the process of fabricating elastomer and electrode layers reproducibly without a need for rigid external frames. We finally characterize the actuation behavior of the fabricated bending DEA fingers and demonstrate their gripping abilities.

## 2. Materials and Methods

### 2.1. Choice of the Elastomer

VHB acrylic elastomer tapes display optimal actuation properties, but they generally require a rigid external frame. Additionally, the thickness of elastomer layers made of VHB tapes is determined via the degree of pre-stretch. These characteristics ultimately limit VHB’s versatility. To overcome these limitations, we have chosen silicone elastomers which are liquid in their uncured form, and thus can be fabricated in the desired dimensions and do not necessarily need a pre-stretch and a rigid frame.

The following equation, derived from the Maxwell stress equation, explains the actuation behavior of an elastomer. Here, $S_{z}$ is the mechanical strain in the direction of the elastomer’s thickness, p denotes the Maxwell stress, Y characterizes the elastic modulus of the elastomer, $\varepsilon_{0}$ represents the permittivity of free space, $\varepsilon_{r}$ stands for the relative permittivity of the elastomer, V indicates the voltage, and z represents the elastomer thickness [15]. Accordingly, a low elastic modulus and a high relative permittivity of the elastomer result in higher deflections.

(1) $$Sz=-\frac{p}{Y}=-\left(\frac{\varepsilon_{r}\cdot \varepsilon_{0}}{Y}\right){\left(\frac{V}{z}\right)}^{2}$$

Among the commercially available silicones, Ecoflex™ 00-10 (Smooth-On Inc., Macungie, PA, USA) has been reported to have the lowest elastic modulus among the Ecoflex™ series [32] and also a reasonably high relative permittivity (3.2 @ 1 Hz) [33]. Ecoflex™ 00-10 is additionally an affordable and commercially available two-component silicone, making it easily accessible, and thus suitable for the active elastomer material of a DEA. Nevertheless, it has a significantly higher viscosity in its uncured form than the other Ecoflex™ products (14 Pa.s compared to 3 Pa.s for Ecoflex™ 00-30) [32]. Since it is challenging to deposit thin layers of highly viscous liquids with the spin-coating technique, we reduced the viscosity of Ecoflex™ 00-10 by adding Silicone Thinner™ (Smooth-On Inc., Macungie, PA, USA) to the silicone compound. According to the datasheet, Silicone Thinner™ can reduce the viscosity of the Ecoflex™ series down to about 55% of their initial value [34]. Therefore, we also considered the thinned Ecoflex™ 00-10 in our characterization experiments. In addition, an ultra-thin passive elastomer layer was required to cover the electrode layers and prevent any risk of electric shock or contamination. Ecoflex™ 00-10, even when it is mixed with silicone thinner, is a very thick compound, thus it is challenging to deposit ultra-thin layers with fabrication methods such as spin-coating. Therefore, we chose Ecoflex™ 00-30 as the passive elastomer material because it has an even lower viscosity. 

For ease of referencing, we call Ecoflex™ 00-10 and Ecoflex™ 00-30, “Ecoflex 10” and “Ecoflex 30”, respectively. Similarly, we call the Ecoflex™ 00-10 mixed with Silicone Thinner™, “Ecoflex 10T”. The letter “T” at the end of “Ecoflex 10T” implies the addition of Silicone Thinner™ to the silicone compound. In the following sections, we characterize and compare the mechanical and electrical properties of Ecoflex 10T, Ecoflex 10, and Ecoflex 30.

### 2.2. Preparation of Uniformly Thin Layers

To deposit thin elastomer layers in a repeatable and reproducible manner, we spin-coated the silicone compound on a polymethyl methacrylate (PMMA) substrate (Modulor GmbH, Berlin, Germany). Emslie et al. developed a mathematical formula to estimate the final thickness of the spin-coated film [35,36]. Accordingly, we selected the spin-coating parameters such as the rotation speed, rotation time, and the amount of the silicone compound on the substrate to obtain the final desired layer thickness h.

(2) $$h=\frac{h_{0}}{\sqrt{1+\frac{2\rho \cdot \pi \cdot W^{2}\cdot h_{0}^{2}\cdot t}{45\eta }}}$$

In Equation (2), $h_{0}$ is the initial thickness of the coating material, ρ is the density of the silicone compound, η is the viscosity of the silicone compound, W is the number of revolutions of the spin-coating machine per minute, and t is the spinning time.

The estimate according to Equation (2) was later verified by inspecting cuts under the microscope before finalizing the spin-coating recipe to achieve the desired thickness. Our studies on the surface roughness showed an arithmetical mean of 0.091 µm for the silicone layers with a thickness in the range of 50 µm to 500 µm and 50 nm for its PMMA substrate. 

### 2.3. Material Characterization

#### 2.3.1. Stress–Strain Behavior

**Figure 2:**
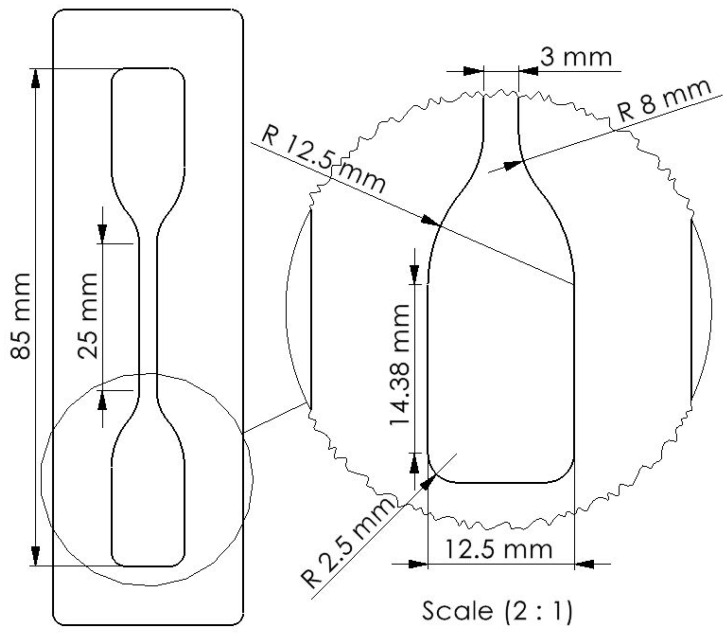
Dog-bone sample molded with Ecoflex™ elastomers for the stress–strain test. The height of the samples is 3 mm.

A mold made of PMMA was used for preparing the samples for the tensile test, as shown in Figure 2. The dimensions are suggested by the standard for the determination of tensile stress–strain properties of elastomers [37]. For the compression test, we molded cylindrical specimens with a diameter of 28.6 mm and a height of 12.5 mm, as suggested by the standard test methods for rubber properties in compression [38]. For each measurement, 3 specimens were tested.

For the tensile test, a universal pull-testing machine (Inspekt table from Hegewald & Peschke, Nossen, Germany), and for the compression test, a rheometer machine (MCR302 from Anton-Paar, Ostfildern-Scharnhausen, Germany) was used. We later stitched the tensile and compressive results together. Since DEAs usually show an unstable behavior and approach breakdown after 33% strain [15], we focused mainly on the lower strain spans.

**Figure 3:**
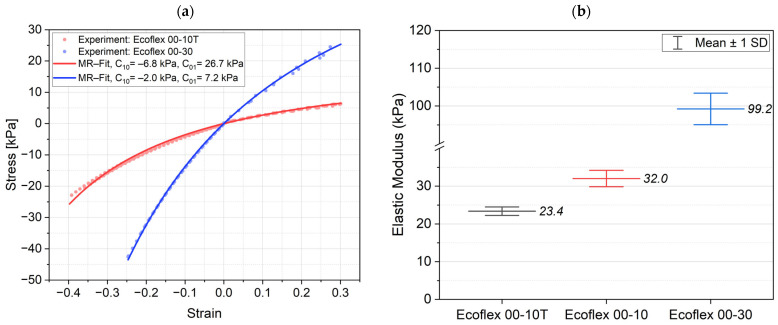
Mechanical behavior of the elastomers: (**a**) the stress–strain curves for Ecoflex 10T and Ecoflex 30 over compressive and tensile strains, and (**b**) comparing the elastic modulus of the silicones to study the effect of silicone thinner on Ecoflex 10.

Figure 3a illustrates the hyperelastic stress–strain behavior of the elastomers (average from 3 specimens per material). We used the two-parameter Mooney–Rivlin hyperelastic model for the curve fitting of the two materials Ecoflex 10T and Ecoflex 30 used for the fabrication of the gripping finger. The stress–strain equation for uniaxial stress is given in Equation (3).

(3) $$\sigma =\left({\left(1+\varepsilon \right)}^{2}-\frac{1}{1+\varepsilon }\right)\;\cdot \left(2\cdot C_{10}+2\cdot \frac{2\cdot C_{01}}{1+\varepsilon }\right)$$

where σ represents the compressive stress, ε shows the engineering strain, and $C_{10}$ and $C_{01}$ are fitting constants.

To investigate the influence of adding the silicone thinner to the Ecoflex 10, we also compared the linear elastic modulus of Ecoflex 10 and Ecoflex 10T. The elastic modulus was obtained by fitting a line to the stress–strain curves in the range of 0 to 10% strain; see Figure 3b. The results indicate that the Silicone Thinner™ decreases the elastic modulus of Ecoflex 10.

#### 2.3.2. Relative Permittivity

To characterize the relative permittivity of the elastomers, we first designed and 3D-printed a housing to mold specimens and conduct measurements according to the guidelines suggested by Carpi et al. [39]. As such, the samples had a low thickness z and large area A to fulfill the recommended ratio for minimizing fringing field effects. This ratio is given as follows:

(4) $$\frac{\sqrt{A}}{z}\geq 100$$

**Figure 4:**
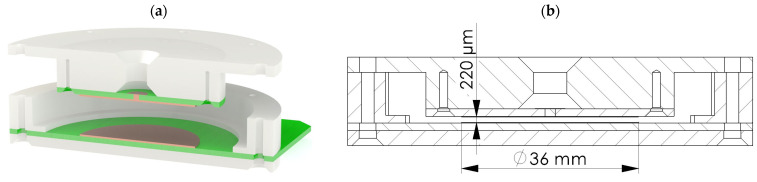
Housing used for molding, curing, and measuring the capacitance of the elastomers: (**a**) the 3D model of the housing, and (**b**) a drawing of the cross-section of the housing illustrating the dimensions of the cured specimen and the measurement electrodes.

To measure the capacitance of the elastomer, the uncured elastomer compound was poured into the housing and filled the measurement space between two copper pads, as shown in Figure 4a. Therefore, we managed to fabricate dielectric layers in the shape of a disk with a diameter of 36 mm and thickness of 220 µm, as illustrated in Figure 4b.

**Figure 5:**
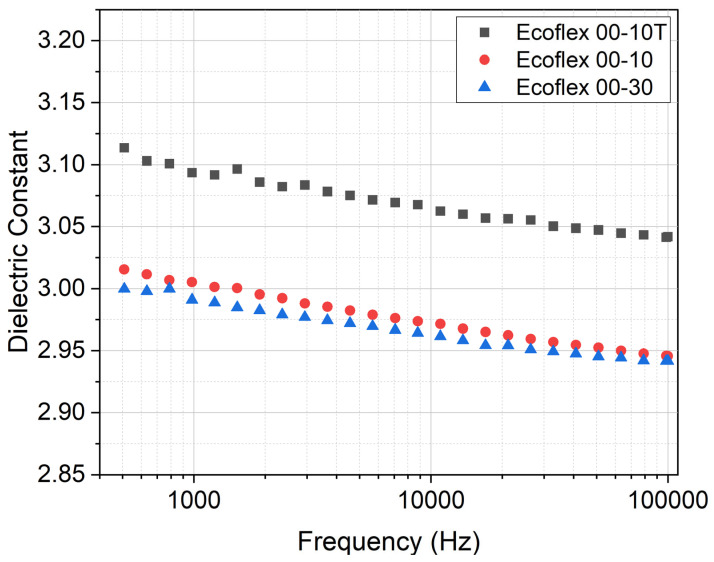
Relative permittivity of the elastomers for frequencies up to 100 kHz.

Finally, we calculated the relative permittivity from the measured capacitance, as shown in Equation (5). Accordingly,

(5) $$\epsilon_{r}=C\cdot \frac{4z}{\pi \cdot D^{2}\cdot \epsilon_{0}}\;,$$

where C is the capacitance and D (=36 mm) and z (=220 µm) are, respectively, the diameter and the thickness of the investigated disk-shaped elastomer. The capacitance values of the samples were measured with an LCR meter (IM3536 from HIOKI, Nagano, Japan) for frequencies up to 100 kHz. For every measurement, we tested 3 specimens. The results, shown in Figure 5, indicate that adding Silicone Thinner™ to Ecoflex 10 slightly increases the relative permittivity which is in favor of achieving higher actuation strains for fast movement.

### 2.4. Electrodes for Multi-Stacking

**Figure 6:**
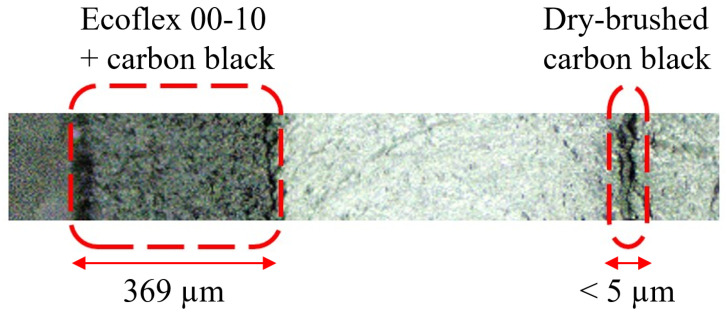
Cross-section of a sample fabricated to compare the electrode layer made of dry-brushed carbon black versus the electrode layer made of a mixture of Ecoflex 10 and carbon black.

As previously mentioned, carbon grease is a suboptimal electrode material to produce multi-stacked actuators. The other conventional method to deposit electrode layers is to mix carbon black with silicone compound and cure the mixture. This is a reliable method to make silicone conductive; however, the resulting electrode layer adds to the stiffness of the actuator, thereby decreasing actuation force and motion. In this study, we dry-brushed carbon black (ENSACO 250 from Imerys S.A., Paris, France) directly onto the silicone layer without using any bonding agent. Shigemune et al. have researched dry-brushing electrodes on elastomer layers [40]. To pattern dry-brushed carbon black powder, we lasered out a negative mask from a PMMA sheet (Modulor GmbH, Berlin, Germany). We placed the mask directly onto the cured elastomer layer and used an ultra-soft cosmetic brush to gently distribute the carbon black particles evenly onto the elastomer surface. The carbon black particles exhibited a strong adhesion to the surface of Ecoflex 10T, and the painted layers remained conductive even under high actuation strains. The profilometry studies on the surface roughness of the electrode layer indicated an arithmetical mean roughness of 0.52 µm for layers with a thickness of less than 5 µm. Figure 6 shows the cross-section of an actuator made for comparing the resulting layer thickness of two different electrodes, one achieved by mixing the elastomer and carbon black and the other one fabricated by dry-brushing carbon black directly onto the cured elastomer layer.

To test the conductivity of the dry-brushed electrodes, we implemented a four-point measurement technique to eliminate measurement inaccuracies caused by contact resistances. The resulting resistivity for a cloverleaf pattern was 0.006 Ω.m per 1 µm of layer thickness, with the van der Pauw correction factor of 0.9914, which matches the range of the resistivity of carbon given in the literature [41].

### 2.5. Design and Simulation

**Figure 7:**
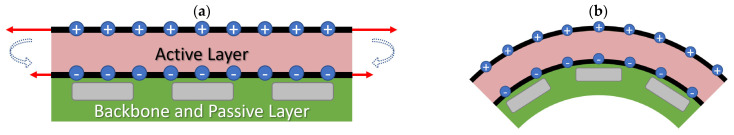
Bending mechanism of a DEA bending finger: (**a**) initial state, and (**b**) final state.

After selecting and characterizing the materials and deposition methods, we fabricated a bending actuator. To do this, we followed the bilayer actuation principle and applied a thick passive layer onto the actuator. The bilayer actuation would result in a biaxial curvature of the finger. Therefore, we embedded stiff backbones into the passive layer to achieve the desired bending direction. Figure 7 shows the bending mechanism, where the red arrows illustrate the relative magnitude of planar displacement, and the curved arrows indicate the resulting bending motion. 

**Figure 8:**
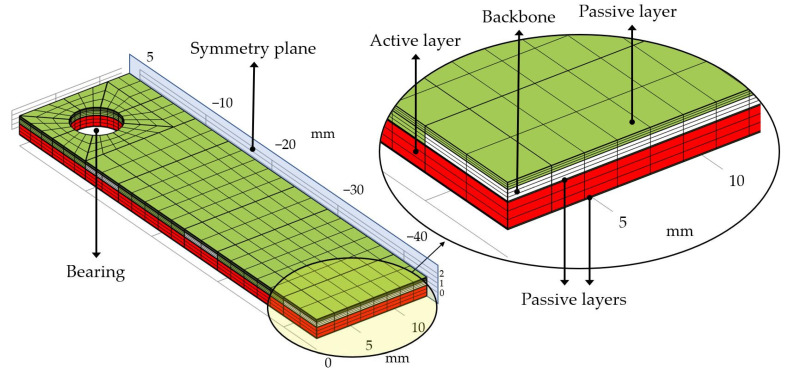
The structured mesh used for simulating the DEA bending fingers. The dimensions of the finger are 45 mm × 25 mm with layer thicknesses of 800 µm for the passive layer (Ecoflex 30), 500 µm for the PMMA backbones, 50 µm for each passive layer (Ecoflex 30), and 550 µm for each active layer (Ecoflex 10T). The electrodes cover the full area excluding the bearing with a 200 µm isolation frame to avoid spark discharge.

A numerical simulation with the commercial software COMSOL Multiphysics^®^ 6.5 was carried out to confirm the design idea and to maximize the bending angle of the finger by adjusting the number of active layers. The electrostatic force was calculated by the electrostatics module (es), and the resulting deformation was calculated by the solid mechanics module (solid). Both modules were coupled by the electromechanical force (eme) coupling to obtain a full electromechanical simulation. Instead of modeling multiple layers for multilayer actuators, we simulated a single layer with adjusted parameters. For an actuator with *n* number of active layers, we simulated an actuator with one active layer with both thickness and actuation voltage increased by a factor of *n*. Therefore, the ratio of the actuation voltage to the overall thickness of the DEA remains unchanged. This method simplifies the simulation process by avoiding excessive modeling effort while maintaining the integrity of the actuator’s physical characteristics, although it also neglects the small influence of the thin electrode layers. The symmetry of the finger design was exploited to reduce the computational effort. A relatively coarse structured mesh with 2332 elements was chosen to avoid excessive computational effort. The structured mesh and the applied mechanical boundary conditions are visualized in Figure 8. The used material parameters are given in Table 1.

**Figure 9:**
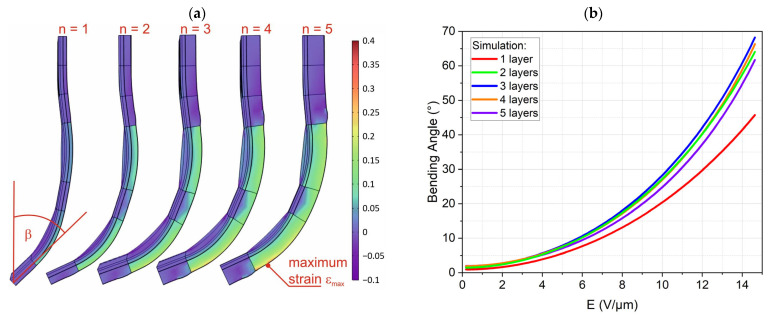
Simulation results for DEA fingers with one to five active layers: (**a**) simulated DEA fingers at 8 kV with maximum strains of 9.7%, 17.7%, 24.1%, 29.3%, and 33.2% from left to right, and (**b**) bending angle *β* vs. electrical field strength.

The simulation was executed as a time-dependent simulation with a voltage ramp of 0.05 kV/s per layer. The simulated finger shapes at 8 kV for two and five active layers with the local strain as color-coded surface are shown in Figure 9a. The maximum strains of about 9.7% for one active layer to 33.2% for five active layers are within the range of strains used for fitting the material parameters, Figure 3a. It is clearly seen that a linear-elastic model would not be suited to model the finger deformation, justifying the effort of using a hyperelastic material model.

We used the bending angle at the tip of the finger as a criterion to judge the finger performance. Figure 9b shows the simulated bending angles versus the electrical field for the DEA fingers with one to five active elastomer layers. The one-layered finger was obviously not strong enough to fully bend against gravity. The fingers with two to four layers showed much higher and very similar bending angles with the highest bending angle at three layers. The finger with five layers showed a reduced bending angle; therefore, higher numbers of layers were not considered.

### 2.6. Fabrication

**Figure 10:**
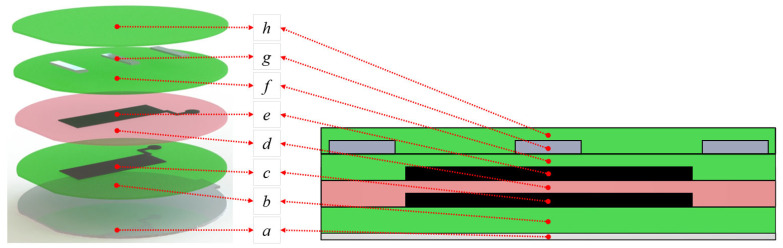
Layer-by-layer fabrication step for manufacturing DEA bending fingers with one active layer: (a) PMMA substrate, (b) first passive elastomer layer, (c) first patterned dry-brushed carbon black electrode layer (25 mm × 20 mm), (d) active elastomer layer, (e) second patterned dry-brushed carbon black electrode layer (25 mm × 20 mm), (f) second passive electrode layer, (g) PMMA backbone structure, and (h) final passive elastomer layer that holds the backbone structure in place.

The layer-by-layer fabrication steps for a DEA gripping finger are depicted in Figure 10. As a first step, a passive layer was deposited on the PMMA substrate with spin-coating techniques. After the passive layer was fully cured in an oven at 80 °C, we patterned the first electrode layer made of carbon black directly onto the passive layer with the dry-brushing method. A PMMA negative mask, lasered out from a PMMA sheet, was used to facilitate the patterning process. Afterwards, the active elastomer layer was spin-coated on the electrode layer and cured in conditions similar to the passive elastomer layer. The second electrode layer was then dry-brushed on the active elastomer layer. For fabricating the multilayer gripping fingers, the steps of spin-coating the active elastomer layer and dry-brushing the electrode layer were repeated consecutively until the desired number of active layers was deposited. The last electrode layer was covered with a passive layer of Ecoflex 30. To implement the backbone structure, stripes of thin PMMA sheets with a thickness of 0.5 mm, length of 25 mm, and width of 5 mm were embedded into the final passive layer. The spin-coating material and parameters for every elastomer layer are shown in Table 2.

**Figure 11:**
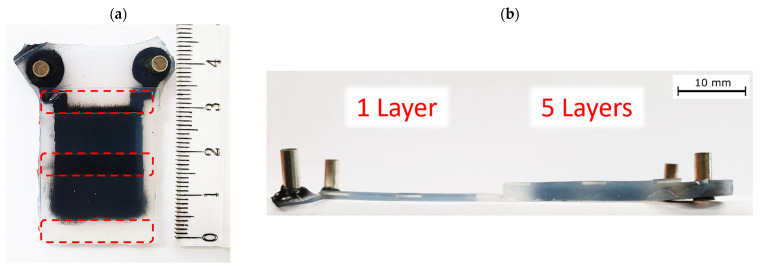
Photographs of DEA bending finger actuators: (**a**) top view indicating the area where the transparent PMMA backbone sheets are placed, and (**b**) side view to compare the actuators with one versus five active layers.

The actuators were later cut in the shape of a bending finger, as shown in Figure 11. The actuators’ weight, dependent on the number of active layers, is presented in Table 3.

## 3. Characterization Results of DEA Bending Fingers 

**Figure 12:**
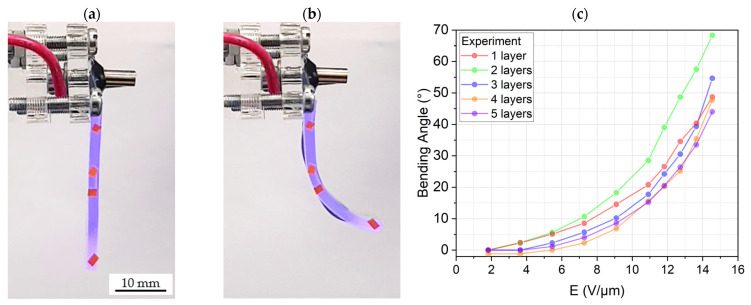
A hanging DEA bending finger with 2 active layers: (**a**) under no actuation voltage, (**b**) bending under 8 kV, and (**c**) bending angle of DEA bending fingers with one to five active layers.

The measurement setup used to characterize the DEAs consisted of a high voltage amplifier (10HVA24-P1 from HVP High Voltage Products GmbH, Martinsried, Germany) and a laser line scanner (scan CONTROL 3002-25/BL from Micro-Epsilon, Ortenburg, Germany). To measure the bending angle of the actuators for voltages from 0 to 8 kV, we applied a ramp voltage signal from zero to the target voltage, and then we ramped the voltage down to zero after holding the target voltage for a certain amount of time. This cycle was repeated for different target voltages, while the bending profile was captured with the laser scanner. The bending angle was obtained by calculating the difference between the angle of the bent profile at the beginning (where the actuator is hanging vertically from its bearing) and the endpoint of the actuator. Figure 12a,b illustrate the bending actuation of a DEA gripping finger with two active layers under 8 kV. The actuation results are shown in Figure 12c.

**Figure 13:**
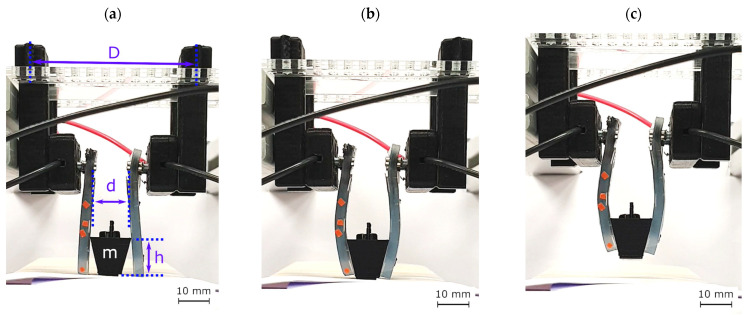
Gripping setup: (**a**) two DEA finger actuators bending towards each other hanging from a linear stage, where d = 11 mm, D = 55 mm, and h = 14 mm, (**b**) DEA gripping fingers grabbing, and (**c**) lifting a basket.

Finally, we assembled a gripping setup by hanging two bending actuators facing each other, as shown in Figure 13a. Each actuator had five active layers and was attached to a linear stage for a vertical up–down movement. To test if the actuators could hold an arbitrary object, we first 3D-printed a basket with a weight of 1.5 g. Afterward, we adjusted the level of the linear stage so that the actuators could reach the basket. Following this, we applied the voltage so that the actuators would bend and grasp the object as portrayed in Figure 13b. We then elevated the linear stage as an act of lifting, as illustrated in Figure 13c. As a final step, we lowered the linear stage again and ramped down the voltage to zero so that the gripping fingers released the basket. The gripping mechanism was only considered successful if all of the gripping steps were performed flawlessly, and the basket did not slip through the fingers. In every gripping cycle, we incrementally added weights to the basket. The gripper grasped and held a maximum weight of 10.3 g, which corresponds to 112% of the DEA fingers’ weight.

## 4. Discussion

The main goal of this work was to demonstrate a frameless DEA gripping finger using a passive elastic layer and rigid backbones to convert the planar motion of a DEA into a uniaxial bending. The choice of materials and fabrication process allowed for high strains at reasonable voltages as well as potential mass production. Using spin-coated Ecoflex 10T as an active material with thin dry-brushed carbon black electrodes led to a five active-layered gripping finger griping and lifting of 10.3 g weight (approximately 100 mN force) successfully. The actuators fabricated with the presented method showed long-term stability meaning that they did not lose actuation performance even after a month from the fabrication time. The characterization of gripping forces achievable with varying numbers of active layers was not conducted in this study due to the complex nature of force generation in practical applications. The gripping force is highly dependent on the geometry of the object being manipulated and the resulting finger shape at the point of contact. Therefore, force measurements taken at artificial positions, such as zero displacement or zero bending angle, would not provide an accurate representation of real-world performance across diverse object shapes. While specific force values are not presented, as a general approximation, the gripping force can be estimated to be about half of the 100 mN in the downward direction. However, this should be considered only as a rough order of magnitude and not a definitive measure of the gripper’s capabilities. Future work may involve a more comprehensive analysis of gripping forces across a range of object geometries and gripping configurations to provide a better understanding of the system’s performance in various practical scenarios.

The bending behavior of the gripping fingers with one to five active layers was also studied. Increasing the number of active layers, on the one hand, increases the bending moment exerted on the gripping finger, which theoretically leads to larger bending angles. However, it also increases the weight and stiffness of the actuator, which counteracts the bending motion in our characterization setup. Therefore, the results, presented in Figure 12, do not show a trend with an increase in the number of active layers. We can see that the maximum bending angle of 68° at 8 kV is for the gripping finger with two active layers.

**Figure 14:**
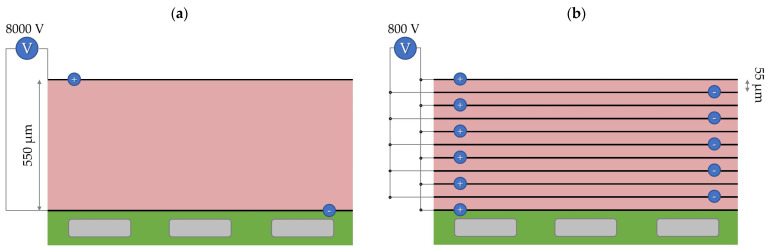
Increasing the number of active elastomer layers without increasing the overall thickness of the gripping finger: (**a**) a gripping finger with one thick (550 µm) active layer driven by 8 kV, and (**b**) a gripping finger with ten thin (55 µm) active layers with the same overall thickness but driven with only 800 V. The bending behavior of both actuators is theoretically the same.

Still, a further reduction in the actuation voltage is desirable. In this work, we did not focus on optimizing the actuation voltage. A total of 8 kV is a high actuation voltage, but it is only required if we aim to actuate thick elastomer layers with 550 µm. In our fabrication method, the mechanical resistance of the electrode layers is negligible compared to that of elastomers; therefore, reducing the thickness of the active layer from 550 µm to about 55 µm (10 times thinner) while increasing the total number of active layers by a factor of 10 would reduce the necessary actuation voltage by a factor of 10 (from 8 kV to only 800 V) according to Equation (1); see Figure 14. It was already demonstrated that layers of Ecoflex 30 can be spin-coated at such a low thickness, but this could be challenging for Ecoflex 10, which has a much higher viscosity in its uncured state. For future works, we will optimize the spin-coating process and/or the material composition for a further reduction in the actuation voltage.

**Figure 15:**
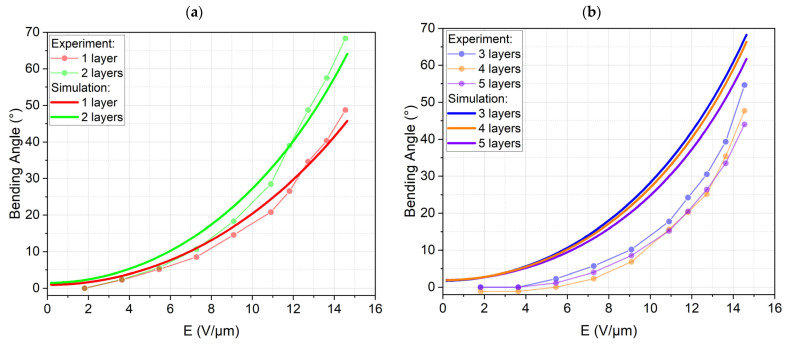
Experimental and simulated bending angles versus electrical field (**a**) for one and two layers, and (**b**) for three to five layers.

A reliable modeling technique is a prerequisite for the future development of more complicated devices with additional degrees of freedom. Simple models based on linear-elastic material behavior and pure in-plane displacement are typically used in the literature [42,43,44]. These models though have limited value for modeling the bending of compound structures with out-of-plane motion. The assumption of linear-elastic materials typically oversimplifies the model. This could be demonstrated by the significantly hyperplastic stress–strain curves of the characterized materials. Therefore, a finite element simulation was used as a modeling tool. Though the effort of conducting a coupled electromechanical simulation of the highly non-linear Maxwell stress actuation is much higher than any analytical model, we expected to benefit from much higher accuracy and deeper insight into the physical effects compared to analytical modeling. This approach was partially successful as shown in Figure 15a. Simulation and experiment for one and two layers agree very well. This is remarkable as no fitting parameters were used to adapt the simulation to the experiment. Figure 15b shows the simulation and experiment for three to five layers. Though the trend is similar, both sets of curves disagree considerably. The experimental bending angles are significantly lower compared to the simulated ones. Several reasons might cause this deviation. Firstly, the electrodes were neglected in the simulation. It was assumed that the electrodes would not contribute to the stiffness of the finger due to their low thickness. Adding more active elastomer layers also means adding more electrodes. The additional layers are also more strained, as thicker fingers lead to higher maximum strains. Neglecting the electrodes in the simulation might, therefore, lead to an error increasing with the DEA layer number. Secondly, the hyperelastic model was fitted to experiments with uniaxial stress in tension and compression. The stress pattern in the finger is, nevertheless, predominately a biaxial stress with compression in thickness direction and tension due to bending. It is possible that the chosen Mooney–Rivlin model does not describe this stress pattern very well. We would again assume that this deviation increases with increasing strain. As a third reason, fabrication tolerances might add up the more layers are applied. This might lead to differences in layer thickness that make the actuator less effective. The contribution of these three effects cannot be quantified with our current knowledge of the materials and processes and will be researched in our future work. Nevertheless, we can summarize that a first step towards modeling these highly non-linear actuators has been conducted, laying the basis for further research.

## 5. Conclusions and Outlook

The objective of this research project was to design and evaluate multi-stacked DEA-based soft gripping fingers that do not require pre-stretching over a rigid frame. To achieve this goal, we first selected and characterized Ecoflex 10 and Ecoflex 30 as our silicone elastomers electrically and mechanically. We then studied and learned that adding Silicone Thinner™ to the uncured Ecoflex 10 compound decreased its elastic modulus from 32 ± 2 kPa to 23 ± 1 kPa, while slightly and desirably increasing its relative permittivity from 3.02 ± 0.03 to 3.11 ± 0.08 (@500 Hz). We utilized the spin-coating technique to deposit thin elastomer layers and implemented the dry-brushing method to apply a carbon black electrode layer with a thickness of less than 5 µm directly onto the elastomer. Carbon black particles exhibited strong adhesion to Ecoflex 10T with an electrical resistivity of 0.006 Ω.m per 1 µm. Afterward, we elaborated on the idea of implementing a backbone structure to translate and direct the planar deflection of the DEAs into a bending motion. In this study, particularly, we performed simulations to model the finger and optimize the number of active layers. We then studied how the number of active layers in a multi-stacked DEA influences the bending behavior of the actuators. The actuators with two active layers showed a maximum bending angle of 68.3°, agreeing with a simulated value of 64°. Simulations for higher numbers of layers, nevertheless, showed a higher deviation from the experiment. Eventually, the developed DEA soft grippers managed to lift and hold a maximum weight of 10.3 g.

For future work, we will focus on several enhancements for the DEA gripping fingers. We will, first of all, minimize the actuation voltage by depositing thinner active elastomer layers while increasing the number of active layers to maintain the overall performance of the gripping fingers. One proposed approach to make ultra-thin layers is to use spray coaters to deposit electrode and elastomer layers. Automating the fabrication process for spray-coating the actuators would be beneficial because we could produce DEAs with a very high number of active layers. We will, additionally, study decreasing the actuation voltage by adding nanoparticles to the elastomer’s uncured compound to increase its dielectric constant which leads to lowering actuation voltages. To improve the backbone structure, we will optimize the backbone material and use lighter and thinner materials and structures.

## Figures and Tables

**Table 1:** Material parameters used for simulating the DEA bending fingers.

Parameter	Ecoflex 00-10T	Ecoflex 00-30	PMMA
Density ρ	1100 kg/m^3^	1100 kg/m^3^	1180 kg/m^3^
Youngs Modulus	N.A.	N.A.	2.9 GPa
Poisson’s ratio	N.A.	N.A.	0.3
Mooney–Rivlin C_10_	−2.0 kPa	−6.8 kPa	N.A.
Mooney–Rivlin C_01_	7.2 kPa	26.7 kPa	N.A.
Dielectric constant ε_r_	3.1	N.A.	N.A.

**Table 2:** Fabrication protocol of the DEA bending fingers.

Function	Material	Spinning Speed (rpm)	Spinning Time (s)	Thickness (µm)
Passive layer 1	Ecoflex 30	2000	20	50
Active layer	Ecoflex 10T	300	15	550
Passive layer 2	Ecoflex 30	2000	20	50
Passive layer on The backbone	Ecoflex 30	N.A.	N.A.	300

**Table 3:** Weight of the actuators with one to five active layers.

Number of Active Layers	Weight (g)
1	1.51
2	2.30
3	3.01
4	3.86
5	4.57

## Data Availability

The original contributions presented in the study are included in the article, further inquiries can be directed to the corresponding authors.
